# Using Machine Learning to Improve Readmission Risk in Surgical Patients in South Africa

**DOI:** 10.3390/ijerph22030345

**Published:** 2025-02-26

**Authors:** Umit Tokac, Jennifer Chipps, Petra Brysiewicz, John Bruce, Damian Clarke

**Affiliations:** 1 College of Nursing, University of Missouri-St. Louis, St. Louis, MO 63121, USA; 2 School of Nursing, Faculty of Community Health Sciences, University of Western Cape, Cape Town 7530, South Africa; 3 School of Nursing and Public Health, University of KwaZulu-Natal, Durban 4041, South Africa; 4 School of Clinical Medicine, University of KwaZulu-Natal, Durban 4041, South Africa; 5 School of Clinical Medicine, Faculty of Health Sciences, University of the Witwatersrand, Johannesburg 2017, South Africa

**Keywords:** South Africa, machine learning, unplanned readmissions, trauma, surgery

## Abstract

Unplanned readmission within 30 days is a major challenge both globally and in South Africa. The aim of this study was to develop a machine learning model to predict unplanned surgical and trauma readmission to a public hospital in South Africa from unstructured text data. A retrospective cohort of records of patients was subjected to random forest analysis, using natural language processing and sentiment analysis to deal with data in free text in an electronic registry. Our findings were within the range of global studies, with reported AUC values between 0.54 and 0.92. For trauma unplanned readmissions, the discharge plan score was the most important predictor in the model, and for surgical unplanned readmissions, the problem score was the most important predictor in the model. The use of machine learning and natural language processing improved the accuracy of predicting readmissions.

## 1. Introduction

Unplanned readmission is a major challenge both globally and in South Africa and is associated with increased health costs and poor outcomes for patients [1,2]. Monitoring the rate of unplanned readmission and understanding the associated risk factors may help institutions improve the quality of care and cost-effectiveness [3]. Identifying risk factors for unplanned readmission may help develop targeted programs to identify patients at risk and reduce the rate of unplanned readmission [4,5]. Although previous studies have used retrospective regression analysis to predict unplanned readmission [6], the development of an algorithm may help prospectively identify at risk patients.

The development of electronic registries over the last two decades has generated large data sets that may be used to generate predictive algorithms [7]. These electronic registries capture data in binary type entries, with distinct numerical values and free text for fields such as patient history. Analyzing binary type entries and distinct numerical values is relatively straightforward using modern spreadsheets and data analysis programs. However, analyzing text entries is more difficult, as the inconsistent nomenclature used to describe key variables are not easily extracted from text. A strategy to address this is the use of artificial intelligence systems such as machine learning, which can allow for the deep mining of large volumes of text and the identification of relationships between text and outcomes [6,7]. Specifically, the use of natural language processing (NLP) in machine learning can be used to analyze free-text data and to convert text to a quantitative format. This may identify correlations between text and outcomes. These factors may be incorporated into predictive models [8].

There has been an increase in the use of machine learning in the health sector in Africa, driven by the need for improved health service delivery [9]. In South Africa, a major obstacle has been the lack of robust electronic health registries and integrated health information systems, particularly within the public health sector, which is compounded by the proposed National Health Insurance [10,11]. This limits the ability to collect, analyze, and utilize health data effectively for informed decision-making, predictive analytics, and the deployment of machine learning models. Therefore, a research gap remains around the predominance of free-text data in such existing systems, rendering it opaque and inaccessible for research purposes.

This project sought to leverage an Natural Language Process NLP machine learning approach to interrogate the Hybrid Electronic Medical Registry (HEMR) database, including free-text entries, with the primary objective of identifying key predictive factors associated with unplanned surgical readmission. The aim was to develop an algorithm that can be integrated into a risk classifier tool to identify patients at risk for unplanned readmission.

## 2. Materials and Methods

### 2.1. Setting

In Grey’s Hospital in Pietermaritzburg, South Africa, a tertiary public health hospital, the Trauma and Surgical Department has maintained an electronic registry, the Hybrid Electronic Medical Registry (HEMR), since 2012. For over a decade, the HEMR has been integrated into the daily workflow of the electronic records of trauma and general surgery patients. The HEMR consists of an electronic menu on which all patient details are entered. This interface captures physiological data and demographic data as distinct fields. It also captures free text documenting each patient’s presenting symptoms, history, clinical plan, operation notes, and discharge summary. To date, the HEMR has accumulated over forty thousand distinct electronic patient entries. These data have been captured with binary yes or no type entries, and fields of free text. Electronic records of trauma and general surgery patients between 2012 and 2022 were included in this study.

### 2.2. Data and Predictors

The outcome metric for this secondary data analysis of a retrospective cohort of patient records were patients who were readmitted unexpectedly to the hospital within 30 days of initial discharge, considered as unplanned readmission [2]. Trauma surgery data included 15,354 patient records from 2012 to 2022, of which 932 patients (6.1%) experienced unplanned readmission. General surgery data included 21,994 patient records from 2012 to 2022, of which 2271 patients (10.4%) experienced an unplanned readmission. Inclusion criteria for this study were limited to individuals aged 18 years and older to focus on the adult population. This exclusion criterion was applied to ensure findings are specifically relevant to adult patients. After removing all patients aged less than 18 years and incomplete data records, 699 trauma patients and 1492 general surgery patients experienced an unplanned readmission and were included in this analysis.

To achieve statistical balance between patients who experienced unplanned readmission and those who did not (other admissions, i.e., patients admitted, planned readmissions, and readmissions after 30 days), a case control design with a stratified random sampling strategy was used. From the data set, ‘other admissions’ were selected (700 patients from the trauma surgery data set and 1495 patients from the general surgery data set) along with the cases of unplanned readmissions. The controls did not experience unplanned readmission and were the same average age as patients who experienced unplanned readmission.

**Figure 1:**
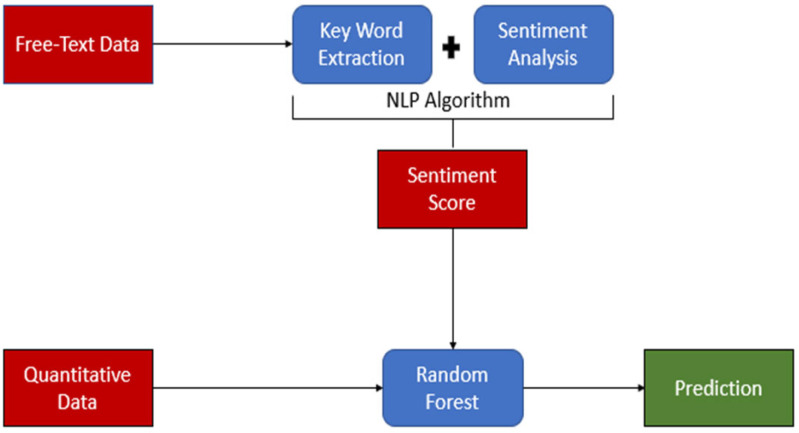
Study flow. The figure illustrates the study flow, highlighting key components using distinct colors: Red represents the data. Blue represents data processing and analysis. Green represents the prediction results. The flow demonstrates the application of Natural Language Processing (NLP) in the study, showcasing the progression from raw data to final predictions.

Available variables included in the analysis were age, sex, diagnosis, diastolic blood pressure, discharge plan, mean arterial pressure, presenting problem, pulse rate, respiratory rate, surgery, systolic blood pressure, and temperature. Presentation and comorbid history, diagnosis, discharge plan, problem, and surgery variables were captured in free text-formed data. To convert qualitative free-text data to quantitative scores, sentiment analysis was used to create a sentiment dictionary using NLP. NLP is a field within artificial intelligence that enables computers to comprehend spoken and written human language [12]. NLP is employed to uncover intricate semantic structures within textual data by leveraging computational algorithms and encompasses aspects distinct from conventional text analysis methods like counting keywords and conducting mapping analysis [13]. See Figure 1 for the overall study flow.

### 2.3. Sentiment Analysis

Using NLP, sentiment analysis was performed to detect and extract subjective information from text data in terms of keywords [12]. Using the R programming language, version 4.0.3, and R packages dplyr [14], string [14], readr [14], and tidytext [15], a sentiment dictionary was created based on the text field in the records of patients who had been readmitted within 30 days. The sentiment dictionary included health condition keywords and their weighted scores, based on their textual frequency, from free-text variables. Keywords were circulated to clinicians within the trauma and surgery department for validation and refinement. Based on the final sentiment dictionary, diagnosis, discharge plan, problem, and surgery texts were scored. These score variables were named the diagnosis score, discharge plan score, problem score, and surgery score. These scored variables were then used as quantitative predictors.

### 2.4. Statistical Analysis

The random forest estimation method was used to predict the risk of patients experiencing an unplanned readmission. Random forest is a machine learning algorithm that combines multiple decision trees to produce a more accurate prediction. The algorithm was implemented using the R programming language, version 4.0.3, and the R package randomForest [16]. Data sets were divided into training and testing sets and a 70:30 split was used, with 70% of data used for training and 30% for testing [17] (Table 1). The training set was used to develop the random forest model, while the testing set was used to evaluate the performance of the model.

A random forest model was used to predict unplanned readmission for each patient in the testing set. The predicted readmission rate was compared to the actual readmission rate to evaluate the accuracy of the model using the following performance metrics to assess the model’s performance: sensitivity, specificity, positive predictive value, negative predictive value, and area under the receiver operating characteristic curve. In addition, to include predictors with highest importance in the model, mean decrease accuracy values were calculated. Mean decrease accuracy is calculated to monitor the impact of each predictor on the accuracy of a random forest model and is a feature of importance measure used in random forest models to assess the impact of each predictor variable on the model’s accuracy. This measure reflects the decrease in model accuracy when a particular variable is randomly permuted while all others are left unchanged. Mean decrease accuracy is computed by comparing the out-of-bag (OOB) error rate of the original model to the OOB error rate after permuting each predictor variable. Variables that result in larger decreases in accuracy when permuted are considered more important. A mean decrease accuracy plot ranks variables based on their importance to a random forest model, with higher values indicating greater importance. This method provides insight into how each variable contributes to a model’s overall predictive performance, considering both the variable’s individual effect and its interactions with other variables in the model.

### 2.5. Ethical Considerations

This study received ethics approval from the university ethics committees (HS22/4/6; UM IRB Review Number 351071) and the KwaZulu-Natal Department of Health (BCA 221/13). All patient data were deidentified to ensure confidentiality and privacy.

## 3. Results

### 3.1. Trauma Surgery Analysis

**Figure 2:**
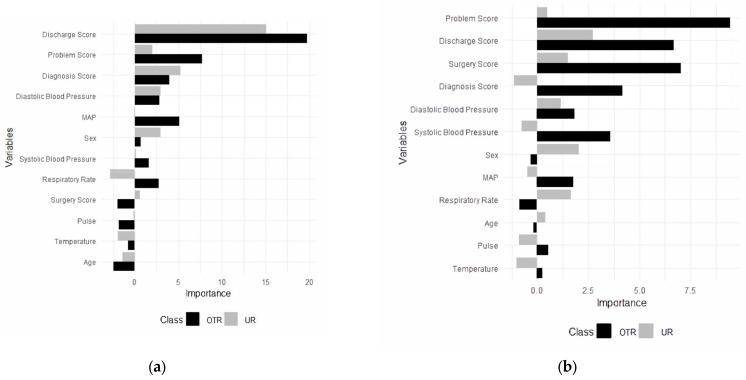
Predictors for unplanned readmissions (URs) and other (OTR) admissions for trauma (**a**) and (**b**) general surgery. OTR = other admissions; UR = unplanned readmissions.

For the trauma surgery model, the final random forest model was built using 12 predictors, including the diagnosis score, the discharge plan score, mean arterial pressure, the problem score, the surgery score, systolic blood pressure, temperature, diastolic blood pressure, sex, pulse, respiratory rate, and age (Figure 2a). Predictors converted from free-text data, except the surgery score, were the most important predictors in the model. Additionally, they were the most important predictors in the model to classify patients in other admissions and unplanned readmissions (Figure 2a).

#### 3.1.1. Accuracy (Trauma Data)

**Figure 3:**
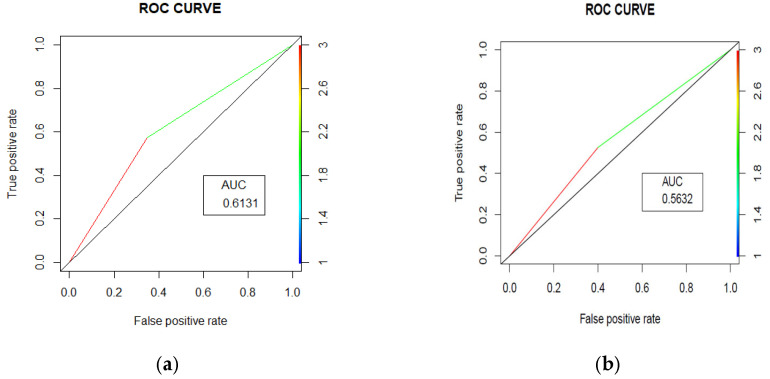
Area under the curves (ROC curves) for trauma (**a**) and surgery readmissions (**b**) prediction.

The random forest model achieved an accuracy of 61.52% (95% CI [56.61%, 66.26%]) with an Area under the Curve (AUC) = 0.631 (Figure 3a). The model’s performance was characterized by balanced sensitivity (65.12%) and specificity (57.51%). The positive predictive value was 63.06%, meaning that when the model predicted an unplanned readmission, it was correct 63.06% of the time. The negative predictive value was 59.68%, suggesting that when the model predicted no unplanned readmission, it was correct 59.68% of the time. The model identified 140 patients correctly as other admission patients, while 111 patients were correctly identified as unplanned readmission patients. On the other hand, 82 patients were incorrectly identified as unplanned readmission patients, and 75 patients were incorrectly identified as other admission patients. The model thus had moderate success in identifying both patients who would and would not have had an unplanned readmission, with slightly better performance in identifying those who would not have had an unplanned readmission.

#### 3.1.2. Key Predictors (Trauma Data)

The discharge plan score was the most important predictor in the model, followed by the problem score, the diagnosis score, and diastolic blood pressure. The discharge plan score emerged as the most crucial predictor, showing substantially higher importance for other admissions and unplanned readmissions compared to other variables. The problem score and the diagnosis score followed as the second and third most important predictors, respectively. Both demonstrated greater importance for other admissions than for unplanned readmissions, indicating they may have been more influential in predicting when a patient was less likely to require unplanned readmission.

### 3.2. Surgery Analysis

For general surgery, a random forest model was built using 12 predictors, including the diagnosis score, the discharge plan score, diastolic blood pressure, mean arterial pressure (MAP), the problem score, sex, the surgery score, systolic blood pressure, pulse rate, respiratory rate, temperature, and age (Figure 2b). Additionally, the most important predictors in the model to categorize patients for both other admission and unplanned readmissions were identified (Figure 2b).

#### 3.2.1. Accuracy (Surgery Data)

The random forest model achieved an accuracy of 56.31% (95% CI [52.94%, 59.63%]), with an AUC = 0.5632 (Figure 3b). The model’s performance was characterized by balanced sensitivity (60.00%) and specificity (52.63%). The confusion matrix revealed that out of 872 total patients, 261 patients were correctly identified non-unplanned readmission patients and 230 patients were correctly identified unplanned readmission patients, while 207 patients were incorrectly identified as unplanned readmission patients and 174 patients were incorrectly identified as other admission patients. This indicates that the model had moderate success in identifying both patients who would and would not have had an unplanned readmission, with slightly better performance in identifying those who would have had an unplanned readmission. The positive predictive value was 55.77%, meaning that when the model predicted an unplanned readmission, it was correct 55.77% of the time. The negative predictive value was 56.93%, suggesting that when the model predicted other admission, it was correct 56.93% of the time.

#### 3.2.2. Key Predictors (Surgery Data)

The problem score was the most important predictor in the model, followed by the discharge plan score, the surgery score, and diastolic and systolic blood pressure for surgery unplanned readmissions. The problem score showed substantially higher importance for both non-unplanned and unplanned readmissions compared to other variables. The discharge plan score and the surgery score followed as the second and third most important predictors, respectively. Both demonstrated greater importance for other admissions than for unplanned readmissions, indicating they may be more influential in predicting when a patient is less likely to require unplanned readmission. Physiological measures such as diastolic blood pressure, systolic blood pressure, and the diagnosis score showed moderate importance, with varying patterns between the different types of admissions.

Demographic factors like sex, MAP, and respiratory rate appeared less influential overall, suggesting that clinical indicators may be more predictive of readmission status than basic patient characteristics in this model. Variables such as age, pulse, and temperature showed notable differences in importance between other admissions and unplanned readmission predictions, potentially indicating specific factors that distinguish between these two outcomes.

## 4. Discussion

Standard statistical analysis such as regression and survival analysis have traditionally been the most widely used techniques for model building, and a recent review suggests that machine learning techniques can improve prediction ability over traditional statistical approaches [18]. Previous studies have indicated that the balanced random forest model outperforms the competition, with a reported sensitivity of 70% and an AUC value of 0.78 for machine learning forecasting of hospital readmissions [19] and all-cause mortality using South African data (AUC = 0.82) [20]. It is important to note that these studies did not utilize unstructured text data as a predictor for hospital readmission. Our project aimed to leverage a natural language processing (NLP) machine learning approach to analyze the HEMR database, including free-text entries. This innovative use of unstructured text data may explain the differences in AUC values observed in our study compared to those reported in other studies. Despite the lower AUC values, our findings underscore the potential of incorporating NLP techniques to enhance predictive modeling in healthcare settings.

Using data captured in real time in electronic health records, we developed and validated a machine learning model to predict the likelihood that patients will have unplanned readmissions within 30 days. Our findings were within the ranges of global studies, with reported AUC values between 0.54 and 0.92 [18,21].

For trauma unplanned readmissions, the discharge plan score was the most important predictor in the model, followed by the problem score, the diagnosis score, and diastolic blood pressure. Patients whose discharge EHRs included keywords (e.g., neurosurgery, trauma, and ICU) selected by clinicians had a higher chance of being readmitted to the hospital compared to those whose discharge EHRs did not include these keywords.

For surgery unplanned readmissions, the problem score was the most important predictor in the model, followed by the discharge plan score, the surgery score, and diastolic and systolic blood pressure. Patients whose problem EHRs included keywords (e.g., sepsis, tumor, and acute) selected by clinicians had a higher chance of being readmitted to the hospital compared to those whose discharge EHRs did not include these keywords.

This is similar to other studies that identified length of stay, disease severity index, being discharged to a hospital, and primary language other than English with increased risk of being readmitted within 30 days [22]. This model provides a focus on data normally captured in free text and adds to the normal predictive values for trauma and surgery readmissions, such as age and comorbidity [23].

## 5. Conclusions

Our research demonstrates a novel approach of harnessing NLP machine learning techniques to extract valuable insights from free-text entries contained within the HEMR. This methodology enhanced the prediction of our model, thereby highlighting the potential for further advancements in healthcare prediction models. The model has moderate overall accuracy, indicating room for improvement, with limitations being that risk scores were developed using patient data from a single institution. The generalizability of our findings will need further validation.

## Figures and Tables

**Table 1:** Data and training data sets.

Area	Train		Test	
	Unplanned Readmission	Other Admissions	Unplanned Readmission	Other Admissions
Trauma Surgery	506	485	193	215
General Surgery	1055	1060	437	435

## Data Availability

Data are unavailable due to privacy or ethical restrictions.
